# The Influence of Executive Functioning on Facial and Subjective Pain Responses in Older Adults

**DOI:** 10.1155/2016/1984827

**Published:** 2016-05-05

**Authors:** Joukje M. Oosterman, Juliane Traxler, Miriam Kunz

**Affiliations:** ^1^Donders Institute for Brain, Cognition and Behaviour, Radboud University, 6500 HE Nijmegen, Netherlands; ^2^Section of Gerontology, Department of General Practice, University Medical Center Groningen, University of Groningen, 9700 AD Groningen, Netherlands

## Abstract

Cognitive decline is known to reduce reliability of subjective pain reports. Although facial expressions of pain are generally considered to be less affected by this decline, empirical support for this assumption is sparse. The present study therefore examined how cognitive functioning relates to facial expressions of pain and whether cognition acts as a moderator between nociceptive intensity and facial reactivity. Facial and subjective responses of 51 elderly participants to mechanical stimulation at three intensities levels (50 kPa, 200 kPa, and 400 kPa) were assessed. Moreover, participants completed a neuropsychological examination of executive functioning (planning, cognitive inhibition, and working memory), episodic memory, and psychomotor speed. The results showed that executive functioning has a unique relationship with facial reactivity at low pain intensity levels (200 kPa). Moreover, cognitive inhibition (but not other executive functions) moderated the effect of pressure intensity on facial pain expressions, suggesting that the relationship between pressure intensity and facial reactivity was less pronounced in participants with high levels of cognitive inhibition. A similar interaction effect was found for cognitive inhibition and subjective pain report. Consequently, caution is needed when interpreting facial (as well as subjective) pain responses in individuals with a high level of cognitive inhibition.

## 1. Introduction

In the general population, the self-report of pain is typically viewed as the golden standard in pain assessment. In dementia, however, these self-reports are limited by the strong cognitive decline accompanying the disease, as it impairs language capacities and thereby the patients' ability to communicate about their pain. Moreover, dementia causes a reduction in abstraction abilities, which reduces the patients' ability to comprehend and thereby use pain scales to indicate their pain. Experts have therefore recently identified pain behaviors as being crucial in order to obtain reliable and valid pain assessments in dementia. Facial expressions form an important part of pain behaviors in the assessment of pain. Facial responses are not compromised by language impairments and, according to some studies, may be less dependent on the desire of expressing pain since facial expression is a rather automatic process [1, 2]. As a result, facial expressions should be less influenced by cognitive decline and are believed to validly indicate pain in patients with dementia [3].

However, although facial expressions of pain are thought to be relatively unaffected by cognitive decline compared to self-report, there is evidence that facial expressions are not completely unrelated to cognitive performance. Keltner and coworkers [4] examined facial expressions in adolescents with internalizing and externalizing problems and emphasized the role of impulse control and inhibition, important prefrontally mediated executive control functions, in the display of emotions. Several other studies examined cognitive and neural correlates of expression suppression, detecting strong associations with prefrontal brain structures and executive control functions [5, 6]. Similar results have been obtained for pain-specific facial expressions: two neuroimaging studies [7, 8] revealed that the suppression of facial pain expressions in low expressive individuals was related to activation in the medial frontal cortex, a structure that is known to be involved in motor and behavioral inhibition [9–11]. This suggests that the degree to which we facially express pain might be related to executive functioning and potentially specifically to inhibitory processes, with low executive functioning leading to higher degrees of expressivity. The reason for this relation might be that we learn in our childhood to control and adjust our facial expressions according to social display rules [12, 13]. According to these social rules, we learn that we should inhibit the facial expression of negative affect, for example, the expression of pain. Depending on our executive functioning, we might be more or less capable of acting upon these rules and inhibit our facial expression of pain. Taken together, several studies suggest that executive functioning, which is strongly dependent on prefrontal cortex functioning, influences the extent to which facial expressions of pain are displayed. Moreover, executive control functions may act as a moderator in the relationship between the level of noxious intensity and the corresponding facial expressions [7].

The goal of the present study was to add to our understanding of how cognitive functioning (especially executive functioning) affects the self-report and facial expression of pain. Studies so far mainly assessed facial expression of pain using the Facial Action Coding System (FACS) [14]. This is a fine-grained analysis system that is, due to being very time consuming, not feasible for use in clinical practice. We therefore decided to investigate the effect of cognitive functioning on observable facial expressions using facial items extracted out of existing observational pain assessment scales. The following hypotheses were tested: (i) first, we expected that a decline in executive control predicts an increase in facial expressions following painful stimulation. (ii) Second, we tested whether this relationship was specific for executive functioning and not an unspecific relation between facial expression and other functions that commonly decline in aging, such as psychomotor speed and episodic memory. (iii) Finally, we examined whether executive functioning moderates the relationship between facial pain expressions and noxious intensity, with those participants with high levels of executive control showing a reduced relationship between facial expressions and stimulus intensity.

As the dementia process induces severe cognitive decline, which hinders both pain report and neuropsychological functions to be reliably assessed, we focused on normal aging adults, as this population is still able to provide reliable pain reports and to undergo a neuropsychological examination. Moreover, we focused on a wide age range, as from the age of 50 years onward a significant decline in cognitive functions, including executive control, can be detected [15, 16]. Therefore, participants from the ages of 50 years and older were included in this study.

## 2. Methods

### 2.1. Participants

Fifty-two older adults between the ages of 50 and 93 years were recruited for this study. Participants were volunteers recruited through advertisements in a local newspaper and through oral advertisement; in addition, some volunteers were acquaintances of the researcher. Education was measured using an ordinal rating scale that ranges from 1 to 7. Here, score 1 represents incomplete primary education, score 2 reflects primary education, score 3 reflects incomplete lower secondary education, score 4 reflects lower general secondary education, score 5 reflects vocational education, score 6 reflects higher general secondary/higher vocational/preuniversity education, and score 7 represents an academic degree [17]. Exclusion criteria were the presence of chronic pain, depression, stroke, a neurological disorder, and daily use of analgesic medication. Furthermore, global cognitive functioning was measured using the Mini Mental State Examination (MMSE) [18]. This test was included to detect the possible presence of severe cognitive problems (score < 24), which was also reason for exclusion from the study. One patient used naproxen and was therefore excluded from the study. All participants gave written informed consent prior to participation. The study protocol was approved by the Institutional Review Board of the Radboud University Nijmegen.

### 2.2. Neuropsychological Examination

All neuropsychological tests were administered in a fixed order. This was necessary to include a fixed delay between immediate and delayed memory testing (see Section 2.2.2.) and to ascertain that this period was filled with the same task demands for all participants. Neuropsychological examinations were conducted by psychology students trained by the principal investigator (JMO). In addition, the administration of the tests was performed in accordance with the standardized instructions as outlined in the manual of the specific tests. The total testing time was, overall, less than one hour.

#### 2.2.1. Executive Functioning

Since executive functioning was our main focus, we employed three tasks to assess this heterogeneous domain. These were the Stroop task, the Digit Span Backward task, and the Zoo Map task. The Stroop task was employed as a measure of cognitive inhibition [19], assessing inhibition of prepotent responses. In short, this test consists of three cards, with each card containing 100 stimuli. The first Word card consists of color words written in black ink, which the participant has to read aloud as fast as possible. The second Color card consists of colored blocks which have to be named as fast as possible. The final Color/Word card contains color names written in an incongruent ink color; here the ink colors have to be named, while reading of the color names has to be suppressed. Participants were instructed to read the words or name of the color as fast as possible. Response times till completion of each card were assessed. The interference score (time needed for the Stroop Color/Word card divided by the time needed for the Stroop Color card) was used for the analyses. It is crucial to note that an increase in the interference score actually reflects worse interference control performance, as participants need more time to complete to complex Color/Word card compared to the time needed to complete the Color card. Working memory was measured with the Digit Span Backward test [20]. Here, series of digits are read aloud to the participants, with the approximate speed of one digit per second, and the participant is requested to repeat these digits in the reversed order. This test starts with 2 digits, which increase in length following successful repetition of at least 1 series. The total number of correctly reproduced series of digits was used as outcome measure. Planning was measured with the Zoo Map, a test that is part of the Behavioural Assessment of the Dysexecutive Syndrome battery [21]. This test consists of an unstructured and a structured part; in both parts, participants are instructed to plan their route through a map of the zoo, visiting a selection of places while bypassing others. While planning the route, participants also need to obey to certain rules (e.g., certain paths can be used only once). In the unstructured part, no information about the exact order is given as participants have to come up with this order themselves, whereas in the structured part the order is explicitly stated. Points are given to places that are visited in the right order, whereas points are deducted in case an error is made. The total score (with a maximum of 16) was used for the current analysis.

#### 2.2.2. Memory Functioning

In addition to these executive function tasks, episodic memory was measured since this function is known to decline with aging as well [22]. Both the Auditory Verbal Learning Test (AVLT) [23] and the Story Recall test (of the Rivermead Behavioural Memory Test (RBMT)) [24] were used for this purpose. The AVLT, measuring memory for unrelated words, consists of a list of 15 words, which are read aloud five times to the participant. Following each presentation, immediate recall is tested, and a total immediate recall score based on the five presentation times is calculated. In addition, delayed recognition was unexpectedly tested after an interval of approximately 15–20 minutes. Story Recall measures memorization of related information that is presented in the form of a story. After an entire story (consisting of 21 distinct elements) has been read aloud by the experimenter, immediate recall is tested. Again, after a delay of approximately 15 minutes, delayed recall is unexpectedly tested.

#### 2.2.3. Psychomotor Speed

Finally, psychomotor speed, a function very sensitive to the age-related decline [25], was assessed using the Word and Color cards of the Stroop test.

#### 2.2.4. Data Processing

For further analyses, standardized scores were calculated for the cognitive outcome measures in order to create cognitive domain scores, so as to reduce the number of statistical tests necessary (which reduces risk of type I error). Hence, an executive domain score (consisting of Stroop interference, Digit Span Backward, and the Zoo Map test), a memory domain score (AVLT immediate recall and delayed recognition, Story Recall immediate and delayed recall), and a psychomotor speed domain score (Stroop Word and Color card) were calculated. Cronbach's alpha was calculated to test reliability of these domains, in order to determine whether it was appropriate to use these domains for the analyses. As previous studies indicated that specifically cognitive inhibition may play a unique role in facial expressiveness, the executive function measures were also examined separately.

### 2.3. Mechanical Stimuli

Perception of noxious mechanical pressure was administered using a Wagner FPX*™* Algometer. Three pressure intensities (50 kPa, 200 kPa, and 400 kPa) were applied in increasing order to both trapezius muscles, yielding a total of six stimuli. These stimulations always commenced on the dominant side. Pressure levels were built up rapidly (within 2 s) and were continued for approximately 5 s. The stimulation intensities were chosen to induce no pain (50 kPa), slight pain (200 kPa), and moderate pain sensations (400 kPa), respectively. In between stimulus applications, pain ratings were recorded, producing short intervals of 10–20 s.

All stimulation sessions were conducted by trained psychology students who also conducted the neuropsychological tests. We used a standardized protocol, and the students performed extensive practice sessions prior to starting the study to assure that they complied to this protocol.

### 2.4. Facial Pain Expression

Facial expressions were video-taped during the mechanical pain test and during a baseline period using a camera that was located in front of the participant at a distance of approximately 1.5 meters. Participants were instructed to maintain focus to a predefined location in front of them, in order to guarantee a frontal view and to avoid talking while pressure was applied. Facial expressions were analyzed offline in time windows of 7 seconds (covering the stimulation period or, in case of baseline trials, the time period before starting the pressure stimulation) using facial descriptors extracted out of existing observational pain assessment scales. This extraction has led to the development of the Pain Assessment in Impaired Cognition (PAIC) metatool [26] as part of a European funded COST action (TD1005) and we used all facial items of this PAIC tool (see Table 1). All facial expressions were rated by an independent rater, trained by the principal investigator (JMO), who was blinded towards the study questions and expectations. In addition, this rater was blinded to study outcomes (e.g., the level of cognitive performance of each participant). To get an indication of interrater reliability, a subset of videos (of 20 participants) was additionally rated by a second rater, namely, one of the psychology students involved in the study. For further analyses, we wanted to select those facial items that are able to differentiate between painful and nonpainful states to form a composite score of pain-indicative facial responses. Following previous approaches [3] we calculated which of the facial items are observed in at least 5% of the 400 kPa trials and which of these items are observed more frequently in response to 400 kPa stimulation compared to baseline (see Table 1). Only these items (they are shaded in grey in Table 1) were summarized to form a pain-indicative facial expression score. Average pain-indicative facial expressions were calculated for each of the stimulus intensities, resulting in one average pain-indicative expression for 50 kPa pressure, one for 200 kPa pressure, and one for 400 kPa pressure. Reliability between the two raters for these pain-indicative expressions, expressed by intraclass correlations (ICC) for each of the pressure intensities, revealed fair agreement between both raters (ICC of 0.43, 0.34, and 0.48 for 50 kPa, 200 kPa, and 400 kPa, resp.).

### 2.5. Self-Report

After each stimulation, participants rated their pain using a 0–10 numerical rating scale (NRS). Average NRS pain scores were calculated for each of the stimulus intensities, resulting in one average NRS pain score for 50 kPa pressure, one for 200 kPa pressure, and one for 400 kPa pressure.

### 2.6. Statistical Analysis

(i) To test the hypothesis that a decline in executive control is associated with increased facial expressions of pain and increased NRS scores, regression analyses were employed, entering the executive function scores as predictor variables and facial expressions or NRS ratings, respectively, as criterion variables. Given that we applied 2 pressure intensities that lay in the noxious range (200 and 400 kPa), analyses were conducted separately for facial and subjective responses to 200 kPa and 400 kPa, respectively, resulting in 2 (NRS scores, facial expression) × 2 (200, 400 kPa) = 4 regression analyses.

(ii) To test whether potential associations are indeed specific for executive functioning, as was suggested by previous studies, we conducted blockwise regression analyses, this time entering memory and speed function in the first block of predictors and executive functioning in the second block. This allows us to test whether executive functioning can add predictive power beyond that already explained by memory and speed performances. Again, analyses were conducted separately for NRS ratings and facial expressions and separately for the 2 noxious intensities.

(iii) In order to examine whether cognition moderates the relationship between pressure intensity and facial and subjective pain responses, repeated measures analysis was employed with pressure intensity (50 kPa, 200 kPa, and 400 kPa) as within-subjects variable and the executive functioning scores as covariates. This analysis was conducted twice, once with the NRS scores as dependent variable and once with the facial expression scores as dependent variable.

Analyses were conducted with SPSS 22 and alpha level was set to 0.05. In case of directed hypotheses, we used one-sided testing.

## 3. Results

Participant characteristics, together with the results from the pain assessment and the neuropsychological examination, can be found in Table 2. For one participant, the facial expressions at 200 kPa pressure intensity could not be rated (due to talking during the stimulation and turning the head downwards). Cronbach's alpha indicated good to excellent reliability of the memory (*α* = 0.86) and psychomotor speed (*α* = 0.91) domains. Reliability of the executive function domain, however, was low (*α* = 0.44), based on which we decided not to use the executive domain score but only focus on the separate executive tests as independent predictors.

### 3.1. Relationship between Executive Functioning and Facial as well as Subjective Responses to Pain (i)

Results from the regression analyses are presented in Table 3. As expected, we found significant associations between executive functioning and responses to painful pressure stimulation. With regard to the NRS ratings, these associations were significant for both mild (200 kPa) and moderate (400 kPa) pressure pain. In contrast, we only found a significant association between executive functioning and facial expression for mild pain stimulation. As can be seen in Table 3, the worse somebody performed in the Stroop interference test (i.e., a higher interference score), the greater the facial expression in response to 200 kPa pressure was. No significant associations were found for facial responses to the moderate pain intensity.

### 3.2. Specificity of the Relationship between Executive Functioning and Facial as well as Subjective Responses to Pain (ii)

Results of the blockwise regression analyses are displayed in Table 4. As can be seen, executive functioning only added explanatory value in addition to memory and speed functioning in explaining variance in facial responses to mild painful stimulation (200 kPa). In contrast, variation in facial responses to 400 kPa and variances in subjective ratings were not better explained by executive functioning than by memory and speed functioning.

### 3.3. Covariate Analyses (iii): Does Executive Functioning Moderate the Relationship between Noxious Intensity and Subjective Responses?

**Figure 1:**
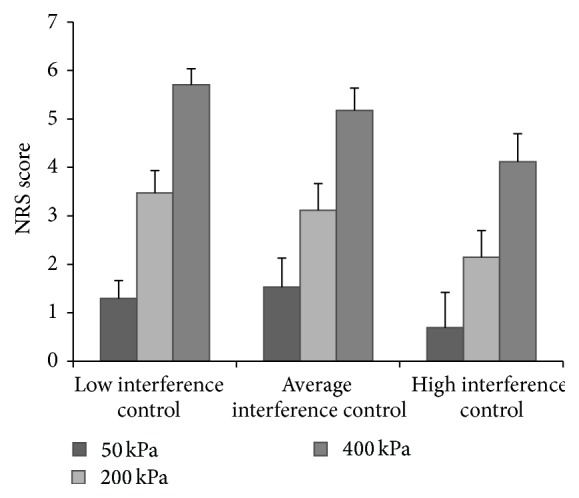
Numerical rating scale (NRS) scores at different pressure intensities of participants with low (*n* = 17), average (*n* = 17), and high (*n* = 17) levels of inhibitory control.

Repeated measures analysis, with pressure intensity as within-subjects variable, the NRS scores as dependent variable, and the executive function measures as covariates, revealed a significant interaction between the Stroop interference score and pressure intensity (*F*(1.32,61.82) = 11.51, *p* < .001, *η* ^2^_*p*_ = .20, and Greenhouse-Geisser corrected). To further examine this interaction effect, three equal-sized Stroop interference groups were created and the analysis was repeated for each group separately (characteristics of these groups are presented in Table 5). For the sake of clarity, we refer to these groups in terms of interference control capabilities. Thus, participants with low interference scores (i.e., less slowing on the complex Stroop Color/Word card) are referred to as having high interference control capabilities. Stated otherwise, these high interference control participants have good executive, inhibitory abilities. Likewise, participants with high interference scores actually have low interference control (i.e., worse executive control), suggesting substantial slowing on the complex Stroop Color/Word card. In all groups, a significant effect of pressure intensity was found, showing increasing NRS scores from 50 to 200 and from 200 to 400 kPa. Results are presented in Figure 1; as can be seen, the groups with average and lowest (i.e., worse) interference control capacities showed comparable increases in NRS scores as the pressure intensity increased. However, the group with the high level of interference control showed a less strong increase in NRS scores as the pressure intensity increased.

The Digit Span Backward and the Zoo test did not interact with the effect of pressure intensity on subjective ratings (all *p* values > .05).

### 3.4. Covariate Analyses (iii): Does Executive Functioning Moderate the Relationship between Noxious Intensity and Facial Expressions?

**Figure 2:**
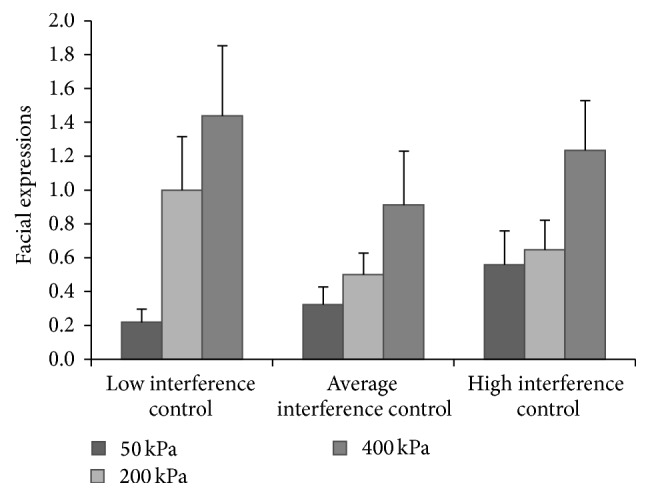
Facial expressions at different pressure intensities of participants with low (*n* = 16), average (*n* = 17), and high (*n* = 17) levels of inhibitory control.

A same repeated measures analysis, now with facial expressions as dependent variable, demonstrated a significant effect of pressure intensity (*F*(1.23,56.58) = 4.37, *p* < .05, *η* ^2^_*p*_ = .09, Greenhouse-Geisser corrected). Repeated contrasts showed that an increase in pressure intensity from 50 to 200 kPa (*p* < .001) and from 200 to 400 kPa (*p* < .05) induced a significant increase in facial expressions. With regard to the interactions between pressure intensity and the cognitive constructs, the interaction with the Stroop interference score (*F*(1.23,56.58) = 7.74, *p* < .01, *η* ^2^_*p*_ = .14, and Greenhouse-Geisser corrected) was significant. Further examination of the facial expressions for each interference control group separately showed a significant effect of intensity in the low interference control group (*F*(1.63,24.40) = 4.76, *p* < .05, and *η* ^2^_*p*_ = .24) but not in the average (*F*(1.14,18.30) = 2.20, *p* = .15,  *η* ^2^_*p*_ = .12, and Greenhouse-Geisser corrected) or high (*F*(1.13,18.11) = 3.38, *p* = .08, *η* ^2^_*p*_ = .17, and Greenhouse-Geisser corrected) interference control groups. As can be seen in Figure 2, the increase in facial expressions from nonnoxious (50 kPa) to slight (200 kPa) and moderate (400 kPa) pain appears to be significantly larger in the group with the lowest (i.e., worst) level of interference control. This increase was less pronounced in the average and high interference control groups.

The Digit Span Backward and the Zoo test did not interact with the effect of pressure intensity on facial expressions (all *p* values > .05).

## 4. Discussion

The present study examined the interrelatedness between cognitive functioning, noxious intensity, and facial expressions of pain in elderly people. Our primary goal was to investigate whether executive function in particular would show a relationship with facial expressions following painful stimulation and if these functions moderated the effect of noxious intensity on facial expressiveness. Moreover, given that subjective pain reports are regarded as being prone to the age-related cognitive decline, in contrast to the more automatically generated facial expressions [1, 2, 27], we expected that cognitive correlates would be more pronounced for these subjective reports than for facial expressions.

Overall, the results showed that variations in subjective and facial responses to noxious stimulation could indeed be explained by executive functioning. The associations were strong for subjective responses, whereas only variations in facial expressiveness to mild noxious stimuli were significantly associated with executive functioning. However, when investigating how specific the associations were for executive functioning compared to the neuropsychological domains “memory” and “speed,” we only found a specific association between executive functioning and facial expressiveness (to mild pain). Here, executive functioning still added explained variance even when controlling for memory and speed performances. In contrast, variance in self-report ratings to noxious stimulation was sufficiently explained by memory and speed performances.

In addition to these regression analyses, we also investigated whether executive functioning might moderate the association between noxious intensity and facial as well as subjective responses using covariate analyses of variance. We found that the interference score as measured with the Stroop test significantly moderated the relationship between pressure intensity and both the subjective pain report and the facial expressions. These interactions indicated that those older adults with better interference control abilities show a less pronounced increase in both the pain report and the facial expressions following painful stimulation, when compared to elderly people with a lower (i.e., worse) level of interference control. A further comparison of these subgroups showed that the only significant group difference was one in age, which supports the notion that it is an age-related decline in inhibition capability that plays an important role in the facial expressions of pain. Moreover, as the interaction effect was isolated to the Stroop task (other executive tasks did not demonstrate an interaction with the facial or subjective expression of pain), we believe that it does not reflect a general effect of age. If it were a general age effect, other executive tests should have also yielded significant interaction effects.

The interpretation of these findings has crucial clinical implications. The relationship between subjective pain ratings and executive functioning appears to be nonspecific: although significant associations with executive control were found, they disappeared after the significant confounding effect of memory and psychomotor speed was included. Hence, regardless of the specific cognitive domain, a general relationship is present where cognitive decline is associated with higher NRS scores. This contrasts with findings for facial expressions, where a unique association with executive functioning was found.

Furthermore, the current study suggests that the level of cognitive inhibition is crucial for the extent to which facial expressions of pain are displayed. A previous study in healthy participants already suggested that cognitive inhibition, as measured with the Stroop interference control score, but not other executive functions such as shifting, working memory, and planning, is associated with experimental pain sensitivity [28]. The current study extends these findings, by demonstrating that interference control may play an important role not only in reporting the severity of pain, but also in the extent to which facial expressions indicative of increasing levels of pain are displayed. More specifically, older adults with high levels of cognitive inhibition may report less pain but also display less facial indicators of pain as the intensity levels increase. How to interpret these finding is currently unclear; it could, for example, indicate that adults with higher levels of interference control can better control their pain, resulting in lower pain reports and less facial pain expressions being displayed, whereas pain levels may be increased in those with lower levels of this control. An alternative interpretation, however, is that people with better interference control are primarily better at inhibiting their pain expressions (both verbal and facial), even though they experience the same level of pain as do older adults with lower levels of interference control. From this perspective, these older adults simply inhibit their explicit expression of pain, but not the* experience of pain *[29], and measuring pain through facial pain expressions might be best applicable in subjects who are not capable of effective inhibition, such as young children and cognitively impaired patients [30]. This is very promising because it indicates that the cognitive decline in patients with dementia (especially the decline in executive functioning) affects the facial expression in a way that makes the facial expression a better indicator of pain and its intensity.

Finally, some other findings deserve attention in the discussion regarding interpretation of facial expressions of pain. First of all, the low interference control group demonstrated increased facial expressions but similar NRS scores compared to the participants with average levels of interference control. This might indicate that this group is specifically unable to inhibit their facial expression to painful stimulation but does not actually experience more pain. Second, given that facial expressions were reduced and did not increase substantially across intensities in the high cognitive inhibition group, interpreting pain based on the facial expressions in adults with high levels of cognitive inhibition could result in an* underestimation* of the amount of pain that is experienced. This is however purely speculative; one potential solution to elucidate this would be to use personalized pain measurements, such as establishing the stimulus intensity of very mild (e.g., NRS score of 2), mild (NRS score of 4), and moderate (NRS score of 6) pain for each individual separately and then to examine whether and how facial expressions change according to the level of interference control.

In contrast to “interference control” the other types of executive functioning (planning and working memory ability) were less strongly related to facial reactivity to pain. It is possible that the commonly assumed heterogeneous nature of executive functioning is also evident in distinct associations with regard to pain outcomes. This suggests that whereas interference control may show an inverse relationship with the level of pain that is reported and facially expressed, this association may be different for other frontal functions. In the existing literature, also indirect evidence for a heterogeneous link between the frontal lobes and pain can be found. Patients with frontotemporal dementia, for example, who normally show severe frontal brain damage, have been shown to display reduced pain awareness as indicated by patients' proxies [31] and increased experimental pain threshold and tolerance levels [32]. Apparently, in these patients reduced frontal lobe functioning is associated with reduced pain, whereas in the current and previous [28] studies, a reversed effect was reported with regard to the relationship between interference control and pain.

Some limitations of the present study need to be addressed. First, participants were instructed to refrain from talking. This might have caused subjects to keep a still face in general and to consciously inhibit facial pain expressions. The fact that the level of facial expressions was generally low might also be related to this point. It is crucial to realize though that the threshold for facial expression of pain is much higher than the subjective pain threshold. That means that individuals just start to facially express their pain once the pain is of moderate or sometimes even strong intensity [33]. Thus, although facial expressions have a very low sensitivity for mild pain experiences, they have a much better reliability for moderate and high pain intensities. One important reason for this is that we learn across childhood to inhibit the expression of negative affect (based on social display rules), including pain [34]. However, since a comparable interaction was found between interference control and the subjectively reported pain, we feel that the observed interaction between interference control and the facial expressions of pain is reliable.

A second drawback of the current study is that the intensities of the applied stimuli were rather low so that some subjects might not have experienced any noteworthy pain. This impression was supported by several participants hesitating or looking doubtful about their pain ratings, especially on the second intensity, as if they were expected to feel actual pain and give higher ratings than on the first occasion but did not really perceive the stimulus as painful. Nonetheless, the fact that we did find a comparable effect of interference control on the increase in pain responses, whether measured by report (subjective) or by facial expressions (objective), supports reliability of our findings.

Third, all stimulation intensities were applied in the same ascending order to prevent that a first stimulation at a high intensity might have an analgesic or even induce a hyperalgesic effect for subsequent lower-level stimulations. Stated otherwise, starting with, for example, 400 kPa stimulation intensity might mask or exacerbate responses to subsequent lower stimulation levels. Although there was a clear rationale for using this fixed order, it might have influenced how participants reacted to the pain. A solution would be to let participants first get accustomed to different pressure intensities, in order to increase reliability of the pain assessment protocol. Regarding the rating of the facial expressions, this was also always accomplished in a fixed order. Hence, the rater may have been influenced by expectations regarding facial expression as the pressure intensity increased. Nonetheless, as the rater was blinded to all study outcomes (e.g., cognitive test results), it is unlikely that expectations of the rater can explain the observed interaction with the interference control score.

Finally, the current study examined only elderly people that were not diagnosed with a neurodegenerative disorder. In order to generalize the results to other populations such as children or patients with dementia, a replication within these populations is necessary.

## 5. Conclusion

The present study indicates that cognitive inhibition moderates the effect of stimulus intensity on pain ratings and facial expressions. Nonetheless, the results also indicate that, in contrast to subjective pain ratings, facial expressions are less likely to be influenced by a general cognitive decline, supporting the clinical utility of these expressions for pain assessment purposes in populations with limited communicative abilities. Future studies are needed, addressing these associations in diverse populations.

## Figures and Tables

**Table 1:** Observation of facial items within the painful trials (400 kPa) in 51 participants. Selection of pain-indicative items was based on frequency of occurrence (>5%) as well as on a more frequent occurrence during pain compared to baseline (effect size *d* ≥ 0.5).

Facial items	Percentage of occurrence^a^	Effect size (Cohen's *d*)
Pained expression	11.8	*d = 0.44*
**Frowning**	**12.8**	**d** = 0.51
**Narrowing eyes**	**17.3**	**d** = 0.59
Closing eyes	5.0	*d = 0.25*
**Raising upper lip**	**16.7**	**d** = 0.53
**Opened mouth**	**33.3**	**d** = 0.85
**Tightened lips**	**19.6**	**d** = 0.58
Clenched teeth	*<5%*	*—*
Empty gaze	50.0	*d = 0.32*
Seeming disinterested	*<5%*	*—*
Pale face	*<5%*	*—*
Teary eyed	*<5%*	*—*
**Looking tense**	**12.8**	**d** = 0.52
Looking sad	*<5%*	*—*
Looking frightened	*<5%*	*—*

^a^Percentage refers to the percentage of occurrence within the painful (400 kPa) trials. Effect sizes for frequency differences between “baseline” and “400 kPa” trials are given. Medium and strong effect sizes (*d* ≥ 0.5) are marked in bold.

**Table 2:** Characteristics, pain NRS, and facial expression scores of the participants.

Variable	*N*	
Age (yrs)	51	66.7 (12.0)
Sex (M/F)	51	26/25
MMSE	51	28.7 (1.4)
Education	51	5 (2)
NRS 50 kPa	51	1.2 (1.6)
NRS 200 kPa	51	2.9 (2.3)
NRS 400 kPa	51	5.0 (2.6)
Facial expressions 50 kPa	51	0.4 (0.6)
Facial expressions 200 kPa	50	0.7 (0.9)
Facial expressions 400 kPa	51	1.2 (1.4)
Stroop Word card (s)	51	55.9 (15.9)
Stroop Color card (s)	51	67.3 (20.1)
Stroop Color/Word card (s)	51	127.5 (95.2)
Zoo Map score	51	11.0 (4.0)
Digit Span Backward	51	5.8 (2.3)
AVLT immediate recall	51	38.6 (11.1)
AVLT delayed recognition	51	27.3 (2.9)
RBMT immediate story recall	51	7.5 (3.4)
RBMT delayed story recall	51	6.3 (3.2)

Descriptive represent means (±SD), with the exception of sex, where frequencies (male (M)/female (F)) are presented, and education where the median score (IQR) is presented. The facial expression scores represent the average number of pain-specific expressions. AVLT: Auditory Verbal Learning Test; MMSE: Mini Mental State Examination; NRS: numerical rating scale; RBMT: Rivermead Behavioural Memory Test; s: seconds; yrs: years.

**Table 3:** Association between executive functioning and facial or subjective responses to noxious stimulation (200 and 400 kPa).

Criterion variable	*N*	Pressure intensity	*β*			*r*	*R* ^2^	*F*	*p*
			Stroop interference	Digit Span Backward	Zoo Map test				
Facial expression	50	200	.575	.054	−.203	.596	.355	8.438	**<.001**
	51	400	−.124	−.233	.123	.223	.050	.820	.244

NRS score	51	200	.299	−.281	.055	.446	.199	3.894	**.007**
	51	400	.321	−.231	.170	.435	.190	3.664	**.008**

NRS: numerical rating scale.

**Table 4:** Specificity of the association between executive functioning and facial or subjective responses to noxious stimulation (200 and 400 kPa).

Criterion variable	Pressure intensity	*N*		Predictor variables	*R* ^2^	Δ*R* ^2^	Significance of Δ*R* ^2^ (*p*)
Facial expression	200	50	Block 1	Memory & speed	.349		
		50	Block 2	Executive functioning	.438	.089	**.044**
	400	51	Block 1	Memory & speed	.005		
		51	Block 2	Executive functioning	.067	.063	.198

NRS rating	200	51	Block 1	Memory & speed	.307		
		51	Block 2	Executive functioning	.335	.028	.293
	400	51	Block 1	Memory & speed	.229		
		51	Block 2	Executive functioning	.281	.052	.184

Results of blockwise regression analyses are presented. NRS: numerical rating scale.

**Table 5:** Characteristics of the three interference control groups.

Variable	Low interference control	Average interference control	High interference control	Statistical test
*N*	17	17	17	—
Age	74.0 (12.3)	67.5 (11.5)	58.6 (6.5)	*F*(2,48) = 9.3, *p* < .001
Sex (M/F)	9/8	11/6	6/11	*X* ^2^(2) = 3.0, *p* = .23
MMSE	28.4 (1.5)	28.6 (1.8)	29.1 (0.9)	*F*(2,48) = 1.0, *p* = .37
Education	4 (1.5)	5 (2)	6 (2.0)	*X* ^2^(2) = 3.9, *p* = .14
Stroop interference score	2.26 (1.85–4.66)	1.71 (1.54–1.84)	1.41 (1.18–1.54)	—

Means (±SD) are presented for age and the MMSE, means (range) for the Stroop interference score, frequencies for sex, and median score (IQR) for education. F: females; M: males; MMSE: Mini Mental State Examination; *N*: number of participants.
